# Correction to “Postacute symptoms 4 months after SARS-CoV-2 infection during the Omicron period: a nationwide Danish questionnaire study”

**DOI:** 10.1093/aje/kwae069

**Published:** 2024-07-04

In the accepted manuscript of the article “Post-acute symptoms 4 months after SARS-CoV-2 infection during the Omicron period: a nationwide Danish questionnaire study” by Spiliopoulos et al^1^ that was posted online ahead of print (November 17, 2023), there were errors that were later corrected for the final version.

When the data collection scheme for the EFTER-COVID questionnaire study was set up, the authors employed a standard validated questionnaire used in one of the study tracks, the Hospital Anxiety and Depression Scale (HADS). The EFTER-COVID survey was launched in haste during the COVID-19 pandemic, and the authors acted in good faith. However, the study design team missed the fact that this scale was copyright-protected and there was a need for permission to use those data. Obtaining permission retrospectively was not possible, and thus deletion of information on HADS from all unpublished and published material was necessary.

Therefore, in the corrected version of this article, the data from the HADS questionnaire results have been deleted, resulting in the removal of 8444 individuals (new study population: *n* = 36 109; old study population: *n* = 44 553).

The study conclusions did not change after the revision, and the changes made to the manuscript were minor. The main changes are that (1) the corresponding 2 mental health symptoms “depression (HADS-D)” and “anxiety (HADS-A)” have been deleted from the list of the 26 symptoms in Figures 1 and 2; (2) 2 references citing HADS have been deleted; (3) Tables S4 and S5, regarding sensitivity analyses of the 2 latter outcomes, have been deleted; and (4) the HADS questionnaire itself has been deleted from Appendix S1.

The authors sincerely apologize for any confusion caused.
